# An Empirical Approach to Analyzing the Effects of Stress on Individual Creativity in Business Problem-Solving: Emphasis on the Electrocardiogram, Electroencephalogram Methodology

**DOI:** 10.3389/fpsyg.2022.705442

**Published:** 2022-03-22

**Authors:** Jungwoo Lee, Cheong Kim, Kun Chang Lee

**Affiliations:** ^1^SKK Business School, Sungkyunkwan University, Seoul, South Korea; ^2^Economics Department, Airports Council International (ACI) World, Montreal, QC, Canada; ^3^Department of Health Sciences and Technology, Samsung Advanced Institute for Health Sciences and Technology (SAIHST), Sungkyunkwan University, Seoul, South Korea

**Keywords:** creativity, stress, ECG, EEG, biopsychosocial model of challenge and threat, cognitive mapping task

## Abstract

In this study, experiments were conducted on 30 subjects by means of electrocardiogram (ECG) and electroencephalogram (EEG) methodologies as well as a money game to examine the effects of stress on creativity in business problem-solving. The study explained the relationship between creativity and human physiological response using the biopsychosocial model of challenge and threat. The subjects were asked to perform a cognitive mapping task. Based on the brain wave theory, we identified the types of brain waves and locations of brain activities that occurred during the creative problem-solving process in a business environment and studied the effects of stress on creativity. The results of the experiments showed significant differences in creativity in business problem-solving depending on whether or not stress was triggered. Differences were found in the time domain (SDNN, RMSSD) and frequency domain (HF, LF/HF ratio) of heart rates, a physiological stress indicator, between the stress group and the no-stress group. A brain wave analysis confirmed that alpha waves increased in the frontal lobe of the brain during creative business problem-solving but decreased when the subjects were under stress, during which beta waves in the brain increased. This study seeks to examine creativity in business problem-solving by studying the effects of stress on human physiological response and cognitive functions in the hope of providing a new and objective interpretation of existing research results.

## Introduction

The source of corporate competitiveness lies in the creativity of the company in question. Creativity refers to the creation of new and useful ideas, products, and services (Amabile and Pratt, 2016) and is considered a critical factor for the success of individuals or organizations (Zhou and Hoever, 2014). In particular, workers with creative problem-solving skills are considered essential resources for business management to respond appropriately to customer demands and improve organizational performance through efficient internal processes (Gotsi et al., 2010).

Given the growing importance of personal creativity in business, there has also been an increase in academic interest in the individual factors that affect creativity (Amabile et al., 2004). These individual factors include personality traits (Batey et al., 2010; Peng et al., 2020), emotional intelligence (Geher et al., 2017), problem-solving style (Basadur et al., 2014), positive emotions (Soroa et al., 2015; Rooij and Vromans, 2020), negative emotions (Baer and Oldham, 2006; De Dreu et al., 2008; Tamir et al., 2015), job stress (Ohly and Fritz, 2010), and emotion control strategies in which individuals either suppress their emotions or positively reappraise them (Costa et al., 2020; Hill et al., 2020). Among the aforementioned factors, job stress—a psychological experience that occurs when workers receive more job demands than they anticipated (Ursin and Eriksen, 2004)—is known to be a negative factor that adversely affects the health and wellbeing of employees in an organization and lowers work concentration and motivation to participate actively (Barney and Elias, 2010). Job stress is caused by a variety of reasons, such as work overload, a lack of freedom, a lack of support from colleagues or superiors, role conflict, role ambiguity, workplace harassment, and time pressure. Employees who experience stress frequently evince low job satisfaction and commitment, and the stress can affect their behavior, for example, through turnover or resignation rates (Parasuraman and Alutto, 1984). As secondary effects, job stress may interfere with the separation of work hours and personal hours, thereby adversely affecting an individual’s wellbeing at home and outside of working hours (Sonnentag and Bayer, 2005).

However, not all stress is bad. Employees experience stress in various situations, for example, while presenting business plans to the company’s high-ranking officials, negotiating salaries, presenting at a conference before numerous attendees, or creating innovative software projects at hackathons (Akinola et al., 2016). In such cases, some people express their opinions logically and in a calm and confident manner while treating these instances as an opportunity to develop and grow, whereas others experience sweaty palms, rapid heart beating, and freezing because of the fear that their demands exceed their abilities or resources.

Since stress functions as a direct factor in eliciting an individual’s psychological and physiological responses, research on the relationship between stress and creativity has drawn substantial attention (Akinola et al., 2019). However, studies have shown conflicting results: some studies have shown that stress has positive impacts on promoting creativity (Byron et al., 2010; Sacramento et al., 2013; Wang et al., 2016), whereas others have shown that stress impairs creativity (Demerouti et al., 2001; Van Dyne et al., 2002). Problem-solving generally refers to the conscious planning and adaptation of processes to attain an outcome that cannot be accomplished quickly; a complicated interaction process of addressing internal and external needs (Heppner and Baker, 1997). Moreover, creative problem-solving is defined as the development of high-quality, novel, and elegant solutions to complex and ill-defined problems (Besemer and O’Quin, 1999). Creative problem-solving not only involves idea generation but also requires processes such as problem identification, information gathering, and idea evaluation (Hunter et al., 2007). However, the research on the effects of stress on the creative problem-solving processes that entail complex cognitive activities in business settings has been considerably inadequate. Thus, this study defines business problem-solving creativity as the creation of as many new ideas and solutions as possible in organizational management, per the concept of creativity as defined by Zhou and George (2001), and treats stress as negative emotions from various job stressors (Giannakakis et al., 2019).

Subsequently, the study aims to explore the impact that stress has on creative business problem-solving and how stress is related to cognitive and physiological processes. The specific purpose of this study is as follows. First, the present research examines the relationship between the physiological responses of the body under stress and business problem-solving creativity. Based on the theoretical background of the Biopsychosocial Model of Challenge and Threat (BPS model; Akinola et al., 2019), which shows the impact on the creative cognitive functions of individuals through stress-induced physiological processes, the study investigates the relationship between stress and business problem-solving creativity through electrocardiograms (ECGs), which measure electrical signals *via* contractions and relaxations in the heart. Second, using electroencephalograms (EEGs), a research method in neuroscience, the impact of stress on the process of solving business problems that require creativity are examined. The effect of stress was confirmed by measuring the differences in the type and area of brain waves in the individuals’ creative problem-solving efforts in the management environment before and after stress.

To the best our knowledge, heart rate variability (HRV) and brain electric dynamics during a business problem-solving task have not been measured in the business context. Thus, it is necessary to study which evaluate the psychophysiological response of individual during a problem-solving task. This is related to the employees in the workplace since they routinely could experience stress. In this regard, we believe that this study can provide a new research frame to explain the effect of stress on employee’s creativity in the business environments.

The study is organized as follows: Chapter 2 comprises a review of the existing literature; Chapter 3 contains the research questions; Chapter 4 details the methodology; Chapter 5 explains the results of the analysis; and, finally, Chapter 6 provides the research findings and implications.

## Literature Review

### The Impact of Stress on Creativity

As stress is known to reduce the attention and increase the reaction times in the industrial production line (Raudonis et al., 2017), the job stress that individuals experience in an organization has been known to adversely affect the wellbeing and health of the employees (Barney and Elias, 2010), negatively affect various areas such as job satisfaction, turnover rate, commitment to the organization, productivity, and creativity (Ding et al., 2019). As it became known that job stress negatively affects not only individuals but also organizations as a whole, research on its association to creativity has gained considerable attention.

According to the aforementioned Challenge-Hindrance Model of Stress (Cavanaugh et al., 2000; Podsakoff et al., 2007), challenge stressors are associated with positive results, whereas hindrance stressors are associated with negative results (Lepine et al., 2005). Per this model, challenge stress promotes creativity in a manner that continuously and actively motivates an individual in relation to the tasks assigned by the organization (Baer and Oldham, 2006). Positive stress, such as time pressure, has been reported to have positive impacts on creativity by encouraging employees’ willingness to learn and incurring proactive behavior in solving problems (Gong et al., 2009; Ohly and Fritz, 2010). Cavanaugh et al. (2000) also classified time pressure, workload increase, and increase in the amount of job responsibility as challenge stressors. Conversely, hindrance stressors occur in situations involving role ambiguity and job insecurity in an organization, and employees perceive the stress as constraint factors on their development and job performance (Lepine et al., 2005; Webster et al., 2011; Ding et al., 2019). When encountering a hindrance stressor, individuals perceive the stressor as something that hinders their personal growth and achievement (Podsakoff et al., 2007). For example, a person who perceives their role as ambiguous and status as unstable might take a relatively passive and timid posture in performing their job duties. Recognizing that the stressors that they are faced with are hindering their growth and development reduces their work concentration and makes it difficult for the individual in question to display focus in terms of creative thinking (Ding et al., 2019). Those who experience stresses from organizational politics and job insecurity become aware that no amount of effort can meet the requirements, thereby resulting in a lack of internal motivation that eventually has a negative impact on their creativity (Fay et al., 2019).

Other studies that explain the negative impacts of stress on creativity through a different approach have indicated that employees experience reduced creativity when they feel that the demands of their work environment are above their expectations or that stress is threatening (Espedido and Searle, 2018) and when they experience emotional fatigue and psychological exhaustion (Demerouti et al., 2001; Van Dyne et al., 2002). According to the cognitive resources theory, stress impairs creativity by limiting the amount of cognitive resources that are necessary for creativity (Vecchio, 1990). By contrast, studies that explain stress as a positive impact on creativity have demonstrated that when employees judge job stress that they experience to be manageable, they use more effective cognitive strategies (Wang et al., 2016) and mobilize their resources effectively by evaluating the stressful situation from positive aspects (Akinola et al., 2019). Some research has also evinced that when employees rate their stress level as low, they might benefit from creative achievements (Byron et al., 2010) and that stress further creates motivation for creativity (Sacramento et al., 2013).

There have been numerous studies on stress and individual creativity to date, as illustrated above; however, the existing research on the effect of stress on creative problem-solving in a business environment still presents conflicting results, and a dearth of research remains in this regard (Akinola et al., 2019; Table 1).

**Table 1:** Previous studies on how stress impacts individual creativity.

Studies	Contents
Fay and Sonnentag (2002); Widmer et al. (2012)	Time pressure positively influences individual intrinsic motivation and wellbeing.
Mumford (2003)	Clear job specifications for individuals help reduce stress that is caused by role conflicts and stimulate work creativity.
Lepine et al. (2005)	Excessive challenge stress forces workers to become excessively involved in their work such that they fail to meet their job requirements and exhaust their energy and resources, thereby seriously impairing their creativity.
Baer and Oldham (2006)	Challenge stress motivates people to seek creative performance and results in higher individual creativity.
Rodell and Judge (2009); Wallace et al. (2009)	When people believe that stressors can be controlled, it helps increase their attentiveness and self-confidence, and they solve problems proactively.
Pearsall et al. (2009)	A certain level of challenge stress stimulates creativity to a certain degree; however, hindrance stress harms creativity mostly when it exists alongside challenge stress.
Binnewies and Wörnlein (2011)	Time pressure has a relationship with creativity in an inverted U shape, and job control moderates the relationship.
Ren and Zhang (2015)	People may be more likely to consider creative problem-solving activities when they strongly feel a sense of responsibility.
Espedido and Searle (2018)	A difficult and challenging goal contributes positively to creative performance.
Fay et al. (2019)	Role-related stress (i.e., role conflicts, role ambiguity, and professional compromise) negatively influences work creativity.
Rostami et al. (2019)	For people such as sales workers, a moderate time pressure serves as an incentive to creative activities.

### A Physiological and Neuroscientific Approach to Stress and Business Problem-Solving Creativity

By scientifically analyzing signals from the human body, physiological and neuroscientific studies can provide a research methodology that allows for a more accurate understanding of human attitudes and behaviors, which traditional methods of self-report and surveys could not identify, and offers varying perspectives and levels of predictability in explaining individual creativity (Akinola, 2010; Blascovich and Mendes, 2010). The present study focuses on creativity in business problem-solving using ECGs and EEGs.

#### Measuring Stress and Creativity Through ECGs

Electrocardiograms comprise a method of measuring the electrical signals generated by contractions and relaxations in the heart. ECGs can analyze heart rate (HR) and heart rate variability (HRV) and indicate the level of stress after observing changes in the autonomic nervous system (ANS) that allows the body to balance changes in the external environment and maintain homeostasis.

Electrocardiogram signal is recorded with electrical impulses which represent the translation into the tracings of line. The wave series is comprised of five waveforms of different characteristics which are recognizable with P, Q, R, S, and T peak. The time between heartbeats is measured in milliseconds, and the main characteristic of ECG signals is that these signals show the distributions of continuous R-R intervals or interbeat intervals.

Heart rate is the most widely used measurement method for measuring stress levels. Stress can be measured by the number of heartbeats per minute (bpm) or by the average RR interval index. Usually, HR increases significantly under stress, and there are almost no claims that the relationship between stress and heart rate is not significant, indicating that it is recognized as a reliable measure (Giannakakis et al., 2019).

Heart rate variability is an indicator that is highly sensitive to stress and is commonly used in stress detection (Palanisamy et al., 2013; Alberdi et al., 2016). HRV reflects the sympathetic and parasympathetic activities of the ANS as it measures the time difference between consecutive heartbeats. In other words, HRV tends to appear low under stress and high under relaxation because HRV indicates that the higher the variation in the normal range is or the more complex the pattern is, the higher the adaptability of the ANS to the stressors is.

Heart rate variability can be divided into a time domain and a frequency domain. In the time domain, the values of the standard deviation of normal to normal R-R intervals (SDNN) and root mean square of the successive differences (RMSSD) in a time-series analysis is known to be parameters that represent the overall stress level well (Alberdi et al., 2016); in the frequency domain, high frequency (HF), LF (low frequency), and the LF/HF ratio are used (Shaffer and Ginsberg, 2017). The LF/HF ratio is an indicator of the balance of the ANS. LF reflects activities in both the sympathetic and parasympathetic nervous systems, but it primarily indicates the activities of the sympathetic nervous system that are responsible for excitation. HF is used as an indicator of the activities in the parasympathetic nervous system that is responsible for relaxation. If the LF/HF ratio rises, the LF is relatively higher, which means the body is excited. In stressful situations, the sympathetic nerves are activated more than the parasympathetic nerves, making it more difficult for the body to cope with stress (Giannakakis et al., 2019). In other words, the LF/HF ratio is to understand whether parasympathetic nerves are less activated in the ANS in the body. In stressful situations, sympathetic nerves are activated; thus, the LF/HF ratio increases. Conversely, in relaxed situations, the arousal (excitement) of sympathetic system calms down; consequently, the LF/HF ratio decreases. Table 2 presents the variations in the HRV indices under stress.

**Table 2:** Changes in heart rate variability (HRV) indices when under stress.

Domain	HRV indices	↑	↓	References
Time domain	SDNN (ms)		●	Blechert et al. (2006); Acerbi et al. (2016)
	RMSSD (ms)		●	Taelman et al. (2011); Acerbi et al. (2016)
Frequency domain	LF (ms^2^)	●		Bernardi et al. (2000); McDuff et al. (2014)
	HF (ms^2^)		●	
	LF/HF ratio (%)	●		Blechert et al. (2006); Acerbi et al. (2016)

*SDNN, standard deviation of the RR intervals; RMSSD, root mean square of the successive differences; LF, low frequency; HF, high frequency*.

Among the existing studies, Wijsman et al. (2013) detected mental stress based on HR, HRV, and other indicators in office-like situations, using various sounds as stressors. De Couck et al. (2019) examined how deep breathing has an impact on decision-making results in a business environment by measuring the HRV of business administration majors. Further, Sharma et al. (2013) asked participants to read texts either with or without stressful content and measured their stress levels using ECGs. Healey and Picard (2005) asked drivers to drive a pre-determined route through open roads in the greater Boston area and measured the level of driver stress using ECGs. Moreover, de Santos Sierra et al. (2011) induced stress by asking participants to give speeches in front of cameras and solve arithmetic problems in virtual reality environments and measuring their stress levels based on HR.

Recently, researchers have used physiological signals using ECG, HRV, etc. to assess and treat various stress or anxiety disorders (Pauws and Biehl, 2015; Akmandor and Jha, 2017; Chen et al., 2017). For example, Ghaderi et al. (2015) and Chen et al. (2017) presented driving-induced stress recognition systems using ECG, GSR, respiratory. Freeman et al. (2018) and Šalkevicius et al. (2019) also presented a framework for stress or anxiety level detection, which is a part of the “Virtual Reality Exposure Therapy (VRET)” using physiological signals acquired from ECG, blood volume pressure (BVP), galvanic skin response (GSR), and skin temperature.

#### Biopsychosocial Model of Challenge and Threat (BPS Model)

Research that links the physiological responses of the human body to individual creativity is nearly non-existent. Furthermore, there is also a lack of theoretical frameworks that can explain the relationship between the two factors. Through the BPS model presented by Akinola et al. (2019), a new way of explaining the impact of stress on personal creativity became possible. Per the BPS model, people assess stress as a challenge when in possession of more resources than the demands from their jobs and perceive stress as a threat when the demands are greater than their resources. Subsequently, their bodies create physiological responses and changes in the ANS to maintain homeostasis; these changes enable them to distinguish stress as either a challenge or threat. Stress is unpredictable and difficult to control, so when people experience stress, the sympathetic–adrenal–medullary (SAM) and hypothalamic–pituitary–adrenal (HPA) axes are activated in their bodies. SAM is quickly activated in situations where it causes a fight-or-flight response, such as being asked difficult questions in front of a large audience at a conference (Akinola et al., 2016; Wadsworth et al., 2019). SAM increases blood pressure and HR by engaging in adrenaline or noradrenaline secretion (Allen et al., 2014). HPA, albeit slower than SAM, continuously causes reactions to secrete the stress hormone or cortisol. Therefore, when exposed to constant stress, the adrenocorticotropic hormone (ACTH) is released in the hypothalamus, which, in turn, leads to a vicious cycle of producing cortisol that inhibits the immune responses of the body (Buske-Kirschbaum et al., 2007). The chronic activation of HPA owing to job stress can lead to increased anxiety, depression (Smith, 2000; Tofoli et al., 2011), and diabetes (Dickerson and Kemeny, 2004).

According to the BPS model, differences in the cognitive functions required for creative achievements occur based on the amount of dopamine produced when responding to stress. Dopamine is known to be essential for regulating cognitive flexibility (Klanker et al., 2013). Because challenges are related to improved cognitive achievements (Mendes et al., 2007), challenge stress activates SAM to secrete the correct amount of dopamine in the prefrontal region (Imperato et al., 1991) and improves the cognitive flexibility required for creativity (Cools and D’Esposito, 2011; Lu et al., 2017). Conversely, threats are related to poor cognitive performances (Kassam et al., 2009), and threat stress causes the activation of SAM and HPA, thereby leading to the excessive secretion of dopamine in the prefrontal region (Zahrt et al., 1997). This eventually hinders the cognitive functioning required to achieve creative achievements (Cools and D'Esposito, 2011).

The BPS model also describes stress from a psychological perspective. In other words, challenges lead to an approach orientation, seeking help from someone, or having positive attitudes such as open-mindedness and self-respect. Physiologically, challenging evaluations result in the activation of SAM, thus leading to an increase in HR and cardiac output as well as a decrease in the total peripheral resistance, which indicates the resistance of peripheral blood-circulating arteries. Threats, conversely, lead to an avoidance orientation and are related to negative motives and negative emotions, such as abandonment, a sense of defeat, fear, and shame. Physiologically, both SAM and HPA are activated, and the HR and total peripheral resistance are elevated, but the cardiac output does not change (Akinola et al., 2019).

In summary, the BPS model explains that when experiencing stress in a business environment, physiological responses occur in the body, and differences in cognitive functions are generated through the process of psychological evaluation on stressors; through these processes, stress either helps or impairs individual creativity. The model proposes methods to measure stress *via* various aspects of physiological responses, including HR, blood pressure, hormones, and eye extension tests. In this study, HRV using heartbeats is used as an indicator of stress.

### Measuring Stress Based on Brain Wave and Creativity Research

Electroencephalograms comprise a non-invasive method of measuring the voltage distribution across the scalp, which is generated by the activity of a large number of neurons. Research that measures brain activity in relation to human creativity using EEGs has a long history. Although more detailed research methods have recently been made possible with state-of-the-art equipment, such as functional magnetic resonance imaging and magnetoencephalography, EEGs are still considered an attractive method because of their relatively low cost and fewer time and space constraints (Stevens and Zabelina, 2019).

According to studies on stress that use EEGs, in stressful situations, alpha wave activities decrease and, because of the need for a high level of cognitive processing effort, beta wave activities increase (Hayashi et al., 2009; Park et al., 2018). Beta waves have been shown to be predominantly active under stress, such as when feeling strong and excited emotions, such as fear (Lindsley, 1952; Jena, 2015). A previous study also showed increments in theta waves during a cognitive tasks such as a stressful chess game (Fuentes-García et al., 2019; Villafaina et al., 2019).

However, in brain wave studies that examined creativity, alpha waves in the relaxation and minimal arousal states have been found to be associated with creative ideation. In a study with 26 participants by (Wang et al., 2019), alpha waves decreased after stress compared to before stress was induced, and creativity level was relatively reduced owing to stress. Lee (2020) as well as Choi and Lee (2016), both conducted research in South Korea and confirmed that negative emotions have a positive impact on business problem-solving creativity. An increase in alpha waves has been shown to occur when performing creative problem-solving tasks in the group where negative emotions were induced.

In terms of changes in oscillations of brain, EEG studies on creativity showed that Alpha waves have consistently been observed over the prefrontal and temporo-parietal cortex during creative ideation (Fink and Benedek, 2014; Schwab et al., 2014) because they may reflect cognitive activity and focused attention (Fink et al., 2009a; Jauk et al., 2013). The neuroimaging studies on neural correlates also have revealed that the inferior frontal gyrus (IFG) and the anterior insula (AI) were the common regions that showed activation for both psychosocial and physiological stress (Kogler et al., 2015).

In a business environment, new ideas are first generated to the fullest through divergent thinking, and convergent thinking is used to evaluate the best options among the ideas to create a creative solution (Althuizen and Reichel, 2016). Studies that examined these creative thinking processes and the properties of brain waves have confirmed the involvement of alpha waves in the creative thinking process. Those who showed high creativity in the divergent thinking task showed a relatively higher alpha band power than those with lower creativity (Fink and Neubauer, 2008; Schwab et al., 2014; Benedek, 2018), and delta waves and theta waves have been shown to decrease (Stevens and Zabelina, 2019). By contrast, those who performed the convergent thinking tasks showed alpha wave activities in the right parietal region when they started to experience insight (Rothmaler et al., 2017), and alpha waves were found to increase in the occipital cortex right before reaching the solution (Jung-Beeman et al., 2004; Table 3).

**Table 3:** Previous brain wave studies on how stress influences individual creativity.

Studies	Contents
Jung-Beeman et al. (2004)	Alpha waves increase in the occipital cortex region just before a solution is achieved in the remote associates test (RAT), measuring the convergent thinking.
Fink and Neubauer (2008)	Those showing a high level of creativity in divergent thinking tasks reveal a relatively higher level of alpha band power than those with a low level of creativity in divergent thinking tasks.
Fink et al. (2009b); Dietrich and Kanso (2010); Luft et al. (2018)	When people work on creativity tasks in a comfortable and minimized arousal state, the alpha waves in their right frontal region are activated.
Schwab et al. (2014)	Those with a high level of creativity in divergent thinking tasks show relatively higher and longer-lasting alpha waves in the frontal region and parietal region.
Lopata et al. (2017)	A relatively stronger alpha wave is shown when performing tasks that require high creativity.
Rothmaler et al. (2017)	Alpha waves can be found in the right parietal region just when people start having an insight in the RAT, measuring divergent thinking.
Schwab et al. (2014); Benedek (2018)	In the context of divergent thinking tasks, alpha band activities are related to a high level of creative performance.
Erickson et al. (2018)	In RAT tasks, people seeking solutions *via* insights activate the beta wave band in their left parietal region, while people seeking solutions *via* analytical approaches activate the beta wave band in the midline frontal cortex. Thus, how people derive creativity can be predicted by utilizing brain waves.
Schwab et al. (2014); Wang et al. (2019)	The alpha wave is shown mainly in the early phase of formulating ideas through divergent thinking; it then decreases and increases again the later phase.
Choi and Lee (2016); Costello and Lee (2020)	Business problem-solving creativity significantly increases in a negative emotion state far more than it does in a positive emotion state.

## Research Questions

Given the scarcity of research on the impact of stress on business problem-solving creativity and the conflicting results obtained thus far, this study sought to investigate the impact of stress on business problem-solving creativity using physiological and neuroscientific research methods. The present study employed the cognitive mapping test and stress-inducing games to map out several new and possible ideas (divergent thinking) in the business environment and analyzed ECGs and brain waves to understand the differences in physiological signals of stress depending on the existence of stress in the creative problem-solving process.

In previous studies, stress has mainly been identified as a factor that hinders creativity by negatively affecting the physiological and cognitive functions of the body. For example, when we experience stress, we feel negative emotions and agitation (Giannakakis et al., 2019), experience psychological pain and dissatisfaction (Soroa et al., 2015; Gu et al., 2018), and have cognitively narrow thinking that thwarts creativity (Baas et al., 2008). Thus, this study assumed stress to be a negative factor in business problem-solving creativity and established the following research questions.

**Research Question 1:** Will business problem-solving creativity be relatively lower in the stress group compared to that in the no-stress group?
**Research Question 2:** Will there be differences in the HRV between the stress group and the no-stress group?
**Research Question 3:** Will there be higher alpha wave activities in the frontal region in the no-stress group and comparatively lower activities in the stress group, and will there be relatively higher beta wave activities in the stress group than in the no-stress group?

## Research Method

### Research Participants and the Groups of Stress

For the experiment in this study, 44 business school students were recruited by posting a recruitment advertisement on the website of one of the Seoul universities. The subjects were enrolled in the 3rd or 4th year of a business school and possessed knowledge about management strategies to solve the business problems of the study. They For the sample size, we referred to EEG experiments in creativity studies that have a between-subject condition, such as Shemyakina and Dan’ko (2007), Grabner et al. (2007), Fink et al. (2009a), and Danko et al. (2009) that had 25–30 participants. Those with preexisting medical conditions and the like, such as high blood pressure, brain damage, and psychiatric treatments, were excluded from the sample. Because the measures used herein were in the Korean language, the sample was limited to domestic university students based in Seoul. To obtain high-quality ECG and EEG data, the participants were asked to abstain from drinking and get enough sleep the day before the experiment.

To produce empirical results for the research questions, stress was induced among the participants through a money game during the experiments. In the money game, participants start playing a computer game with a certain amount of money; the participants earn more money if they win the game and lose money upon losing the game. Both stress and no-stress groups played the money game. Stress was induced among the participants in the stress group by making them lose money. Conversely, the participants in the no-stress group were given a reward of making more money from the original money. During the experiment, the participants were equipped with experimental EEG and ECG equipment. For a valid cross-group comparison, experiments were conducted by randomly assigning 18 participants to a no-stress group and 18 to a stress group. However, six participants, whose data had excessive noise owing to considerable movement during the experiment and to the phenomenon sensor separation, were excluded at the data cleaning stage because their data were not suitable as physiological data in the study. In total, 30 participants were included in the data analysis, with 15 in the no-stress group and 15 in the stress group. The demographic properties of the 30 participants were 20 males (66.7%) and 10 females (33.3%). The average ages were 25.1 for males (SD = 1.744), 24.3 for females (SD = 1.767), and 24.8 for all the participants (SD = 1.763). The participants were given gift certificates for their participation in the research. This study was approved by the institutional review board of the university to which the researchers belong.

### Experiment Procedure

**Figure 1:**
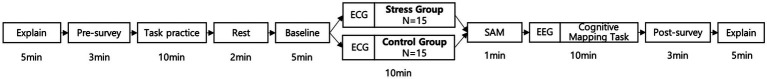
Procedures of the money game experiment.

**Figure 2:**
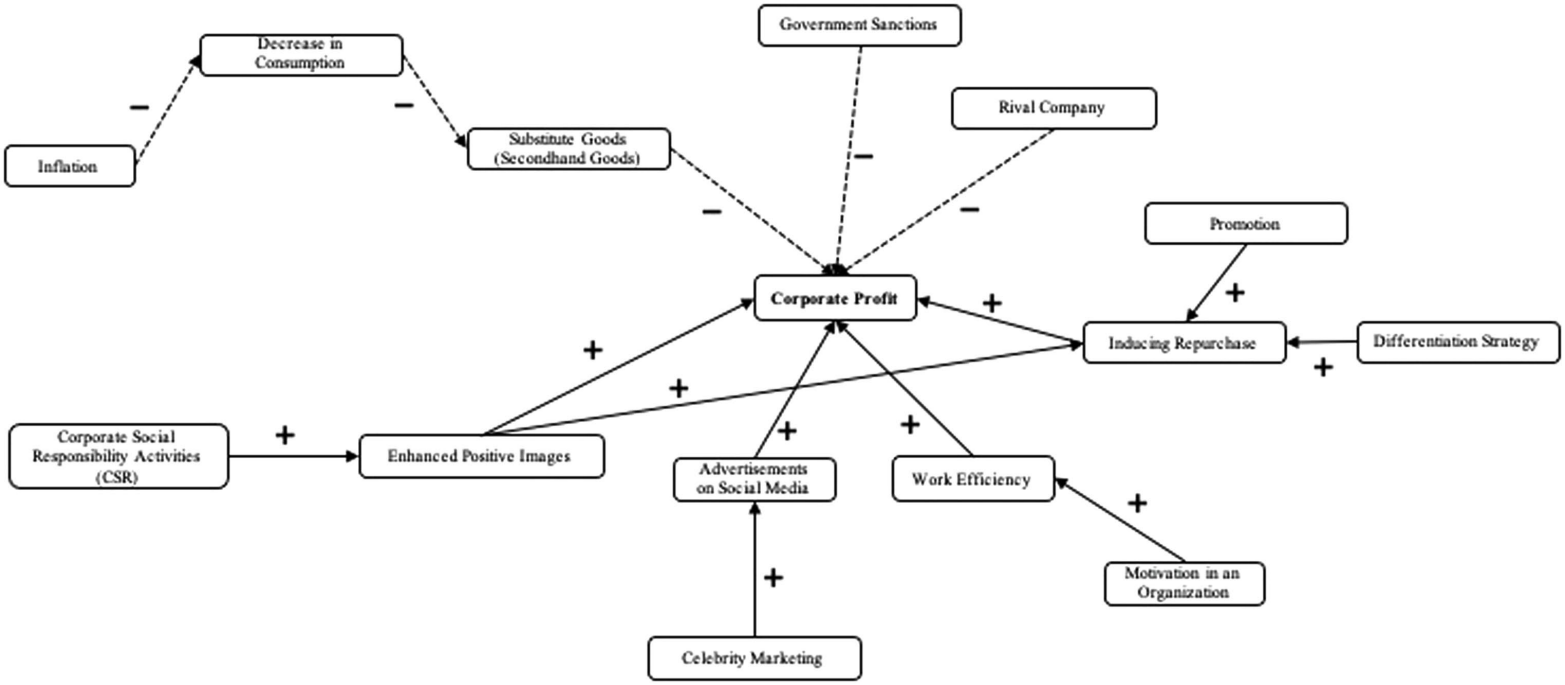
An example of a cognitive mapping test illustrating the cause-effect relationship between concepts (nodes) of a particular problem.

The experiment was carried out at a university’s laboratory in Seoul that was equipped with laboratory monitors, computers, and ECG and EEG equipment; noise was blocked. The procedure of the experiment, as shown in Figure 1, was as follows. The experiment was conducted through nine steps for a total of 50 min. (1) After the participants entered the laboratory, the general contents of the experiment were explained to them. The participants were informed that the researchers were attempting to understand their health and psychological states and would use ECGs and EEGs to measure physiological signals. The agreement of the participants to participate in the study was double-checked. (2) The participants were given instructions on how to operate the keyboard for the cognitive mapping test and stress games that were for measuring business problem-solving creativity. (3) The participants were asked to rest in a comfortable position for 2 min. (4) ECG and EEG sensors were attached to the participants to obtain physiological signals. The ECG sensors were attached based on the bipolar limb leads: the + pole on the left wrist and the − pole on the right wrist. Further, 14 EEG channels were attached to the scalps of the participants, and baseline signals were obtained while the participants maintained a comfortable position for 5 min. (5) The participants played the money game, which was designed to induce stress, for 10 min. (6) The self-assessment manikin was carried out for a manipulation check. (7) The participants were asked to perform business problem-solving creativity tasks. The ECGs and EEGs were measured for 5 min when signal distortion was minimized by asking the participants to describe the concept nodes of the cognitive map; the participants were asked to draw the cognitive map for 5 min while listening to the recording of their voice listing the concept nodes. Using ALMind 1.71 (Eastsoft, South Korea), a cognitive map was created by drawing the concept nodes and connection lines. An example of a cognitive map drawn by one of the participants is shown in Figure 2. (8) A survey was conducted to collect demographic data. (9) Finally, the purpose of the experiment and the measures used in the experiment were explained in detail to the participants.

### Experiment Instrument

#### Electrocardiograms

The BIOPAC MP-150 (BIOPAC Systems Inc., CA, United States) ECG module was used to obtain ECG signals. The signals measured in the hardware were filtered and preprocessed with the low-pass filter 1 Hz and the high-pass filter 35 Hz provided by AcqKnowledge 4.2 (BIOPAC Systems Inc., CA, United States); through these processes, R peaks were extracted. Preprocessed data derived HRV-related values in time and frequency domains using Kubios HRV ver. 3.4.2 (Kubios Oy, Finland). Finally, a statistical analysis was performed using IBM SPSS Statistics 21.

#### Electroencephalograms

**Figure 3:**
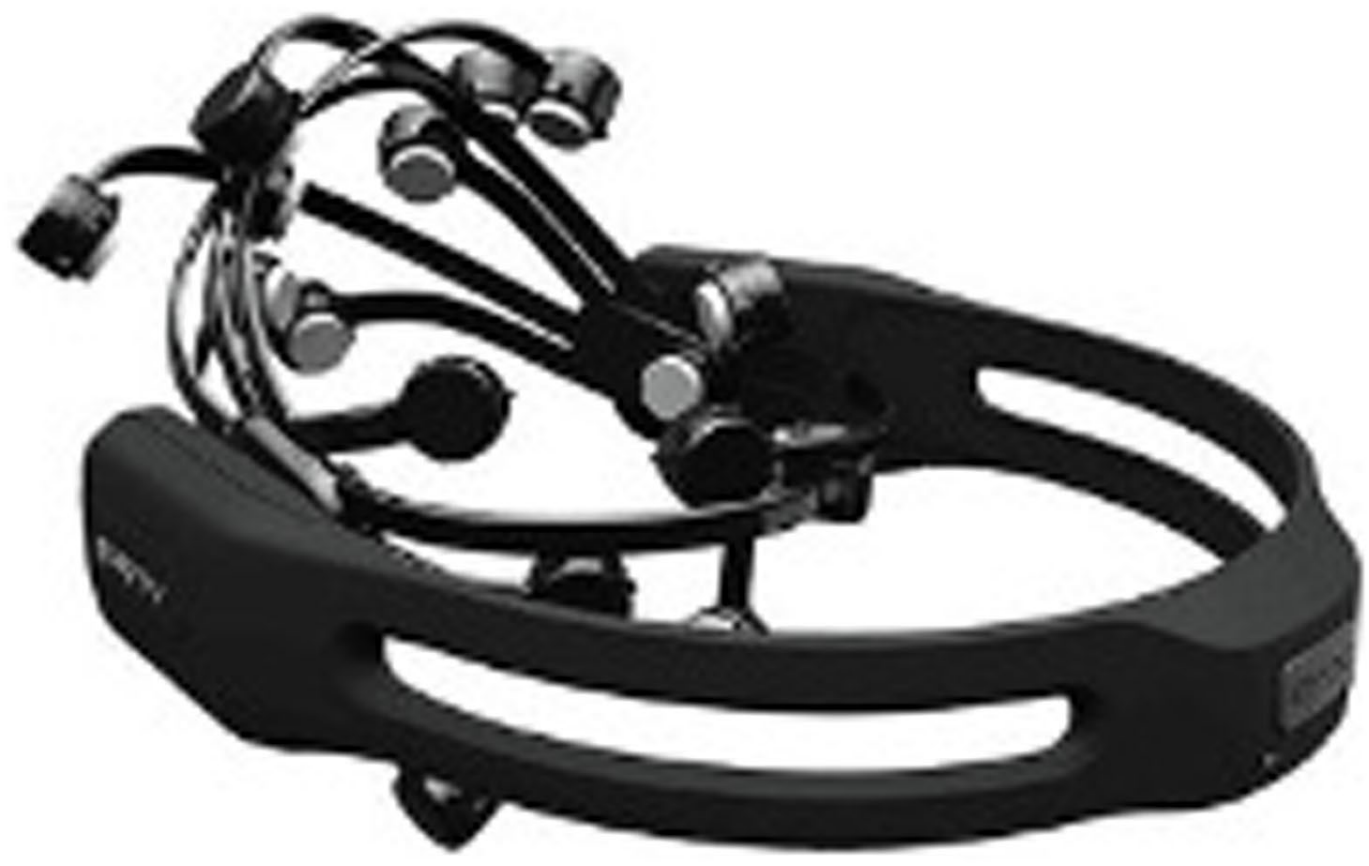
Emotiv EPOC+ (wireless EEG headset).

An EPOC+ (Emotiv Systems, Inc., CA, United States) 14-channel wireless headset was used to measure the brain waves of the participants. The headset EEG device is shown in Figure 3. In compliance with the 10–20 system, the internationally recognized electrode placement method (Homan et al., 1987; Mendoza-Palechor et al., 2019), the electrode attachment locations to measure brain waves were a total of 14 channels, including AF3, AF4, F3, F4, F7, F8, FC5, FC6, P7, P8, T7, T8, O1, and O2. The two channels of common mode sense (left) and driven right leg (right) were each located on the mastoid process of the left and right sides of the brain and used as a reference. All EEG data were sampled at a frequency of 128 Hz. The EEG data were collected using the TestBench software of Emotiv Systems, Inc.; the preprocessing and analysis of EEG data were carried out using Curry 7.0 (Components Neuroscan, Inc., NC, United States). The baseline correction of the data was set to constant. Band pass filters and an independent component analysis were applied between 0.1 and 30 Hz to eliminate noise in EEG signals that was caused by the muscle and eye movement of the participants. Performing a fast Fourier transformation of the EEG time-series data, in each frequency band of delta (0–4 Hz), theta (4–8 Hz), alpha (8–13 Hz), beta (13–30 Hz), and gamma (30–50 Hz), allowing for the relative band power parameters for each frequency to be derived. IBM SPSS Statistics 21 was utilized for the statistical processing of the data.

#### Stress-Inducing Game

**Figure 4:**
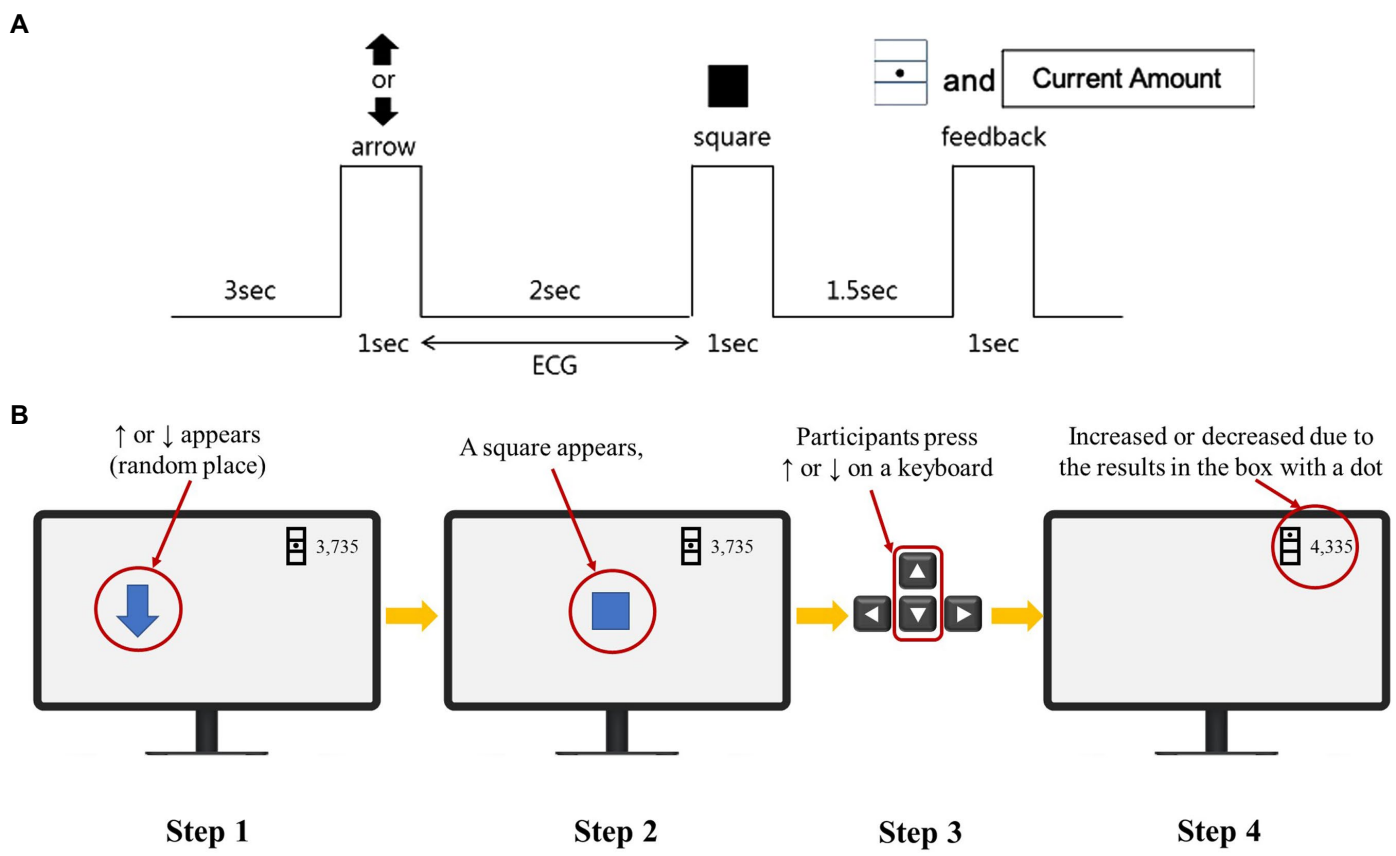
**(A)** Money game experiment paradigm. **(B)** Money game experiment example.

Previous studies have used various methods for stress inducements, such as the International Affective Picture System (Hosseini and Khalilzadeh, 2010), sound (Wijsman et al., 2013), stress situation templates (de Santos Sierra et al., 2011), reaction time measurement game (Zhang, 2017), arithmetic problems (Jun and Smitha, 2016), chess game (Fuentes-García et al., 2019; Villafaina et al., 2019), cyber game (Williams et al., 2000;Loudon et al., 2017), delivering speech (Šalkevicius et al., 2019) and movies (Kolodyazhniy et al., 2011). However, in the present research, a money game was developed to induce stress based on the reaction speed game used by Sobotka et al. (1992). Figures 4A,B show the experimental paradigm and an example for the money game. In the game, the participants saw the arrows (↑ or ↓) appear on the monitor; then, when a square (■) shape appeared, the participants had to immediately press one of the arrow keys on the keyboard that has the same direction as the arrow on the screen showed. If the response speed met certain criteria, the participant was compensated in the game; if the participants failed, they lost some money in the game. Arrow directions were randomly presented, and feedback was given for each attempt. The stress group received feedback that their response speed was slow even no matter how fast they reacted, while the no-stress group received feedback that their response speed met the criteria. The feedback was provided using three boxes on the screen, with the middle box representing the criteria (no money acquisition), the top box (money acquisition), and the bottom box (money loss); a dot was marked on a box in each case. The feedback showed both the speed and the amount of money of the participant. Ultimately, the stress group was set up to lose the game money, whereas the no-stress group was set up to gain some game money; for instance, if the initial amount is 2,000, the stress group will more likely reach the amount of less than 2,000 and the no-stress group will eventually acquire more than 2,000 as their final score. Before the game began, the participants were motivated by being told that they could earn the money that they gained during the game. The game was created using E-Prime 2.0 software (Psychology Software Tools, Pittsburgh, PA).

#### Cognitive Mapping Test

The cognitive map, first introduced by Axelrod (1976), is a test that illustrates the cause–effect relationship between each concept (node) of a particular problem in a drawing by using and connecting lines that represent directions and +/− signs that define the relationship. It is a method that links the causal relationships between multiple concepts (nodes) using arrows and represents in a drawing (Mpelogianni and Groumpos, 2018). This method has the advantage in that the relationships between nodes, which affect the target nodes, can be easily understood and can be measured objectively. The scenarios required to create the cognitive maps were written with reference to (Lee and Kwon, 2006; Lee, 2020). The key point of the scenario was that the participants, who were in charge of a SPA brand strategy, had to establish a management strategy that could maximize corporate profits by increasing the sales, which had been reduced owing to an uncertain business environment and a decline in domestic and foreign demands. Figure 2 shows an example of the cognitive mapping test.

### Evaluation Method and Manipulation Check

Cognitive map scores were calculated based on creativity factors such as creative fluency and originality (Baas et al., 2013). For creative fluency, the participants were given one point each to the concept node and connection line in their drawings; for originality, the participants were given one point if the node they derived was a unique one among all the nodes presented by the other participants. With reference to the previous studies using the cognitive mapping test (Lee and Kwon, 2006; Lee, 2020), we averaged the score of creative fluency and originality into one score.

The self-assessment manikin was used to indicate how much stress the participant had immediately after the game (Lang et al., 2008). The self-assessment manikin was developed to measure affective valence and arousal. As discussed by Giannakakis et al. (2019), stress represents emotionally negative valence and positive arousal; therefore, the present study used the self-assessment manikin for a manipulation check on stress. The self-assessment manikin was used to indicate the good and bad valence of emotion from 1 (unpleasant) to 9 (pleasant) and the degree (arousal) of emotion from 1 (mild) to 9 (intense). The measurements showed that the no-stress group had an average valence of 5.933, with a standard deviation of 1.033, and average arousal of 4.867, with a standard deviation of 0.640, whereas the stress group had an average valence of 4.133, with a standard deviation of 0.639, and average arousal of 6.333, with a standard deviation of 0.724. The results of the t-test to see the differences in the self-assessment manikin between the two groups showed that both valence (*t* = 5.738, *p* < 0.000) and arousal (*t* = −5.880, *p* < 0.000) were significantly different between the groups.

**Figure 5:**
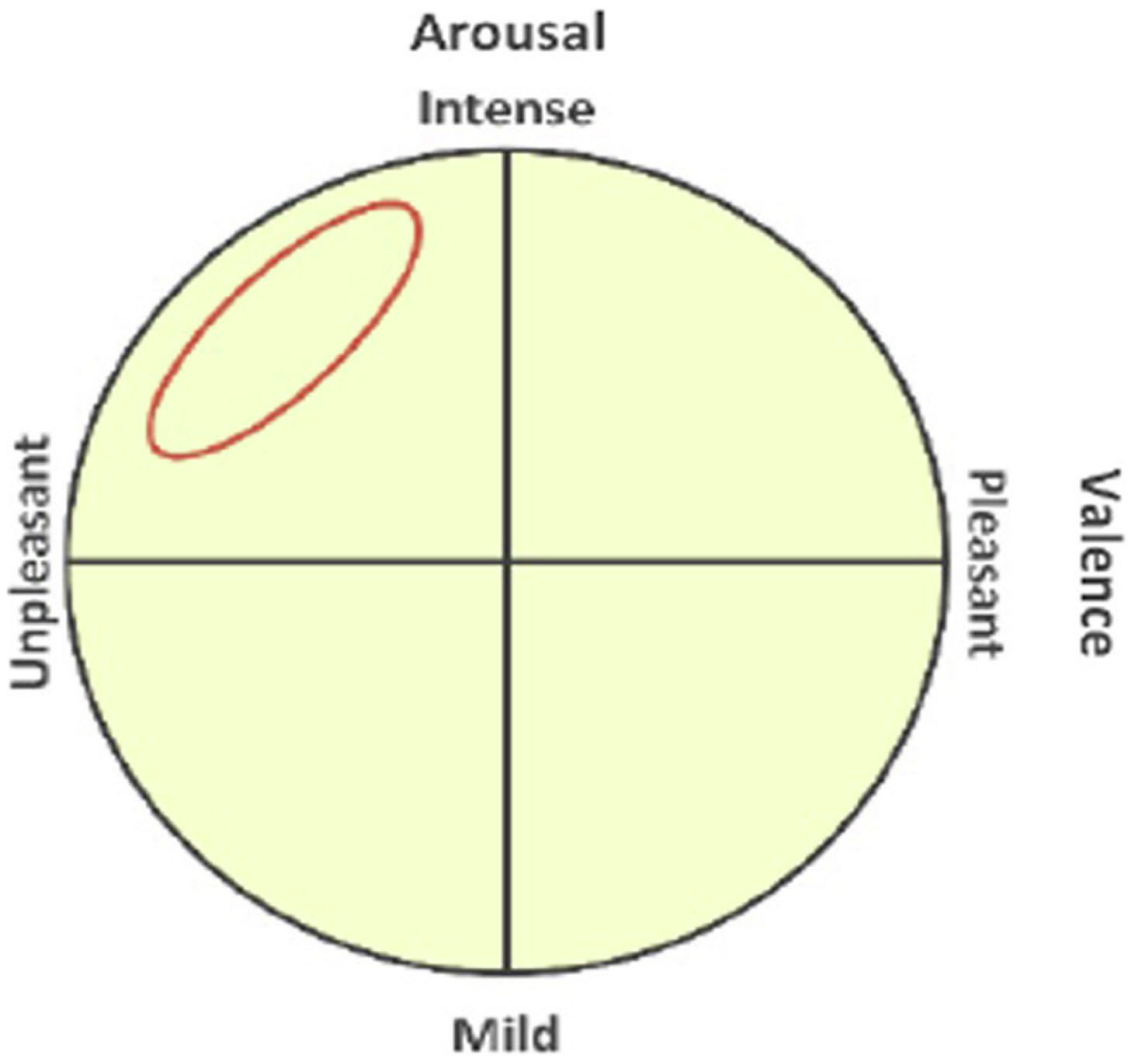
Two dimensions of emotion and stress (Giannakakis et al., 2019).

**Figure 6:**
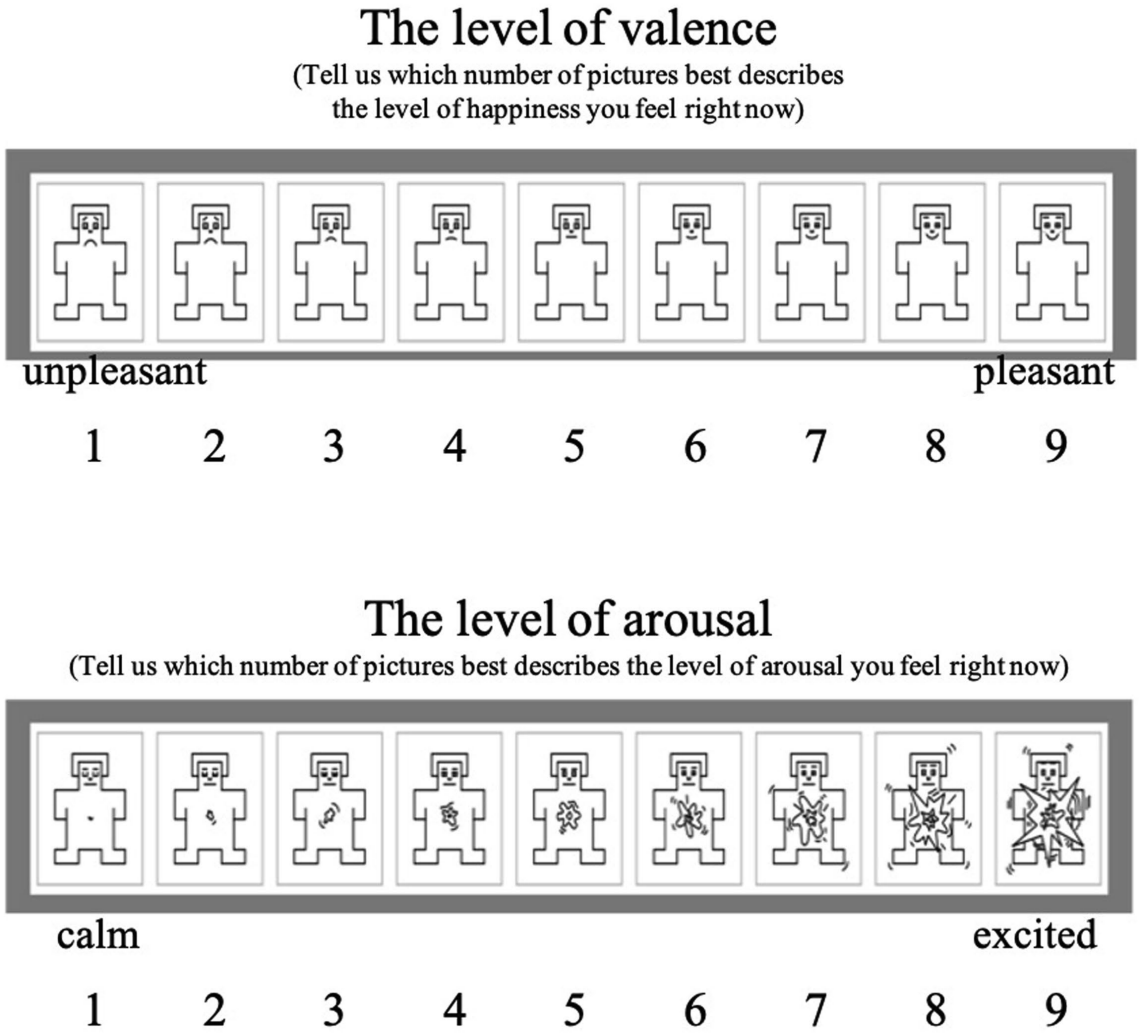
Self-assessment manikin for a manipulation check on stress.

Figure 5 is a graphical representation of the space in which stress was located in the segmented emotion domain; Figure 6 is a representation of the self-assessment manikin used herein.

## Research Results

### (Research Question 1) The Analysis Result of the Effect of Stress on Business Problem-Solving Creativity

Research Question 1 aimed to understand the differences in business problem-solving creativity depending on the presence of stress. To answer the question, an independent sample *t*-test was conducted. The differences were found to be statistically significant (*t* = 2.175, *p* < 0.05) based on a significance level of 0.05. Therefore, it can be said that there are differences in business problem-solving creativity depending on stress; the no-stress group had an average of 36.73 points, and the stress group had an average of 31.73 points, indicating the relatively higher average score of the former compared to the latter (Table 4).

**Table 4:** Cognitive map score: mean differences according to stress (*N* = 30).

Groups		Cognitive map score			*t*	*p*
		*N*	Mean (*M*)	Standard deviation (SD)		
Stress or No-stress	No-stress group	15	36.733	7.648	2.175	0.04^*^
	Stress group	15	31.733	4.559		

* *p < 0.05*.

### (Research Question 2) Differences in Physiological Responses Due to Stress

Research Question 2 anticipated that the physiological responses of the body would differ depending on the presence of stress. In other words, it was assumed that there would be differences in HRV between the stress and no-stress groups; specifically, the stress group was expected to show significant differences in the indices in time and frequency domains of HRV. An independent sample t-test was conducted to determine the mean differences in the HRV of the two groups according to the stress manipulation; the results showed significant differences in the standard deviation of the RR intervals (SDNN; *t* = 3.057, *p* < 0.01), RMSSD (*t* = 2.758, *p* < 0.05), HF (*t* = 3.004, *p* < 0.01), and LF/HF ratio (*t* = −4.867, *p* < 0.000). LF was higher in the stress group, but it was not statistically significant (Table 5).

**Table 5:** Mean and standard error for baseline heart rate variability (HRV) measures during the money game.

Indices		No-stress group	Stress group	*t*	*p*
Time domain	SDNN (ms)	59.067 ± 13.554	40.879 ± 18.632	3.057	0.005^**^
	RMSSD (ms)	35.7333 ± 12.127	24.1333 ± 10.875	2.758	0.01^*^
Frequency domain	LF (ms^2^)	1418.706 ± 303.999	1703.866 ± 446.259	−2.045	0.5
	HF (ms^2^)	1128.796 ± 574.713	658.987 ± 191.207	3.004	0.008^**^
	LF/HF ratio (%)	1.452 ± 0.511	2.771 ± 0.913	−4.867	0.000^***^

* *p* *< 0.05;*

** *p* *< 0.01;*

*** *p < 0.000*.

### (Research Question 3) Differences in the Degree of Brain Wave Activation Between the Groups When Performing Business Problem-Solving Creativity Tasks

In Research Question 3, it was assumed that when performing business problem-solving creativity tasks, the brain waves and activated brain regions would be displayed differently depending on the presence of stress. In other words, the activity of alpha waves in the frontal region was expected to be high in the no-stress group and relatively low in the stress group. Moreover, the beta waves of the stress group were predicted to be relatively higher than those of the no-stress group. The analysis showed that the no-stress group had somewhat higher alpha wave activities in the F4 and AF4 regions of the right frontal region compared to the stress group at a significance level of 0.05 (*t* = 2.284, *p* < 0.05). In addition, the stress group showed relatively higher beta wave activities in the F7 region of the left frontal region and the F8 region of the right frontal region compared to the no-stress group at a significance level of 0.05 (*t* = 3.011, *p* < 0.05). The analysis for Research Question 3 is shown in Table 6.

**Table 6:** Comparing brain waves and activated regions during a cognitive mapping test.

Brain wave	Region	Stress vs. No-stress group	Mean	Standard deviation	*t*	*p*
Alpha	F4	No-stress	0.174	0.059	2.284	0.019^*^
		Stress	0.116	0.063		
	AF4	No-stress	0.160	0.045	3.011	0.005^*^
		Stress	0.102	0.055		
Beta	F7	No-stress	0.119	0.054	3.096	0.004^*^
		Stress	0.187	0.058		
	F8	No-stress	0.154	0.067	2.116	0.026^*^
		Stress	0.202	0.039		

* *p < 0.05*.

## Discussion and Conclusion

### Discussion

The main purpose of this study was to explore which cognitive and physiological processes were related to stress in relation to creative business problem-solving. In detail, the relationship between the physiological responses of the body and business problem-solving creativity according to stress was examined using ECGs. Through a neuroscientific research method, the impact that stress has on a business problem-solving process that requires creativity was also investigated using brain wave analysis techniques. To this end, an experiment was conducted using a money game in which the participants were divided into stress and no-stress groups and performed business problem-solving tasks. The analysis showed that there were differences in business problem-solving creativity depending on the presence of stress and that the level of business problem-solving creativity in the stress group was relatively low compared to the no-stress group. Following the experiment, from a physiological standpoint, differences in the participants’ HRV, which indicates the presence of stress, were confirmed through the data extracted from the ECGs depending on whether stress was or was not experienced. The brain wave analysis showed that contingent on the presence of stress, differences existed in activated brain waves and regions when performing business problem-solving creativity tasks. The detailed analyses of the experiment are as follows.

First, it was expected that stress would have a negative impact on business problem-solving creativity. The analysis showed significant differences in business problem-solving creativity depending on whether stress was induced, and the level of business problem-solving creativity in the stress group was relatively low compared to the no-stress group. This supports previous research that indicated that stress hinders the cognitive effort and thinking process required for creative problem-solving. Positive moods and negative moods, such as stress, in individuals affect creativity through flexibility and persistence, respectively, according to the Dual Pathway to Creativity Model (Baas et al., 2008; De Dreu et al., 2008; Nijstad et al., 2010). In other words, positive moods motivate individuals to perform tasks, which helps generate more ideas and solutions through a cognitive flexibility path. Conversely, negative moods, such as stress, engender avoidance motivation, thereby leading to a cognitive persistence path. This path enhances creativity by allowing systematic and analytical efforts within few specific domains (Baas et al., 2013). Cognitive flexibility paths positively affect divergent thinking, whereas cognitive persistence paths positively affect convergent thinking. In this context, it can be interpreted that the participants in the no-stress group herein had increased cognitive flexibility owing to positive emotions, thereby resulting in their comparatively better results in the divergent thinking process (cognitive mapping tests) than the participants in the stress group. Although conflicting results exist in the extant research on the effects of stress on creativity, the present study derived results from an experimental environment that required cognitive activity to perform business problem-solving creativity tasks and thus provides empirical evidence on how stress affects cognitive creativity (Choi and Lee, 2016).

Second, it was anticipated that there would be differences in the physiological responses of the body depending on the experience of stress. Namely, differences would exist in the HRV between the stress group and the no-stress group; the stress group was expected to have significant differences in the indices in the time and frequency domains of HRV. When the HRV between the two groups was compared, the stress in the stress group was higher, as expected. Further, SDNN and RMSSD in the time domain were significantly lower in the stress group, which can be taken to mean that as the HRV decreased, it became difficult for the body to resist stress. In the frequency domain, it was found that the HF, which indicates the state of the parasympathetic nerves associated with relaxation, was significantly lower in the stress group. Furthermore, the differences in the LF/HF ratio between the two groups were significant. Therefore, the sympathetic nerves were excited in the stress group, thus increasing the LF/HF ratio; because these nerves were more excited in the stressful situation than in the previous condition, it became physically difficult to deal with stress.

According to the BPS model by Akinola et al. (2019), physiological reactions occur in the human body when experiencing stress in an organization. Subsequently, the SAM and HPA are activated, and blood pressure and heartbeats increase (Allen et al., 2014). Based on the BPS model, the present study explored the stress level of the participants using the HR-related stress indicator (HRV). To summarize Research Questions 1 and 2, the participants in the stress group showed relatively higher levels of stress and lowered cognitive mapping scores than those in the no-stress group. This may be because the SAM and HPA were simultaneously stimulated, resulting in a relatively reduced cognitive function. However, the no-stress group scored lower in the stress indices compared to the stress group and had higher cognitive map scores, possibly because cognitive flexibility was enhanced owing to the activation of SAM. Thus, the attempt herein to explain the effects of stress on business problem-solving creativity in relation to bodily physiological responses and cognitive functions has provided new and objective interpretations with respect to the previous research.

Third, it was anticipated that differences would exist in the activated brain waves and regions between the groups when performing business problem-solving tasks. It was also expected that alpha wave activities in the frontal region would be high in the no-stress group and relatively low in the stress group. Beta waves were expected to be relatively high in the stress group compared to the no-stress group. The analysis showed that alpha waves had relatively high activities in the right frontal region (F4, AF4 regions) in the no-stress group, while beta waves evinced relatively high activities in the left and right frontal region (F7, F8 regions) in the stress group. These results support the findings of previous studies wherein the brain waves related to creativity were alpha waves, and excitation and arousal were highly associated with beta waves (Fink et al., 2009b; Schwab et al., 2014; Jena, 2015). Namely, the cognitive mapping test used in the present study to measure business problem-solving creativity is a creativity task related to divergent thinking; thus, the study aligns with previous research in which divergent thinking was highly correlated with alpha waves among brain waves (Benedek, 2018).

Moreover, beta waves responsible for cognitive functioning in the brain were activated in the frontal region, which can be interpreted to mean that the money game used herein to induce stress was effective and that cognitive efforts were actively made in performing creative problem-solving tasks in a state of emotional arousal, namely, stress (Hayashi et al., 2009; Gola et al., 2013; Jena, 2015). Furthermore, in the no-stress group, alpha waves were increased, and beta waves were decreased more than before when performing cognitive mapping tests. In the stress group, both a decrease in alpha waves and an increase in beta waves were evident when performing the test. To summarize the results of Research Question 3, the participants in the stress group were hindered in creative ideation by stress as they performed the tests in the aroused state, resulting in relatively poor creative performance.

The theoretical implications of the present research are as follows. First, the study shed light on the negative effects of stress on creativity in a management environment from a physiological perspective. It physiologically explained the impact that stress has on business problem-solving creativity by using the BPS model, which describes how stress has a positive or negative effect on individual creativity in a business environment by generating differences in physiological responses and cognitive functions. Second, the study broadened the scope of interpretation of the existing literature by considering the effects of stress on business problem-solving creativity through a brain wave analysis—a neuroscientific method. Finally, previous stress and creativity studies that used paper-based assessments and surveys are limited in terms of internal and external validity or have a socially desirable bias and are also limited in relation to measuring or predicting the exact state of the participants (Stevens and Zabelina, 2019). Thus, because of its use of physiological and neuroscientific approaches, the current study is expected to complement the limitations of previous research and further provide a framework for objective interpretation (Josephs et al., 2006).

In addition, the practical implications are as follows. For employees in an organization, stress is a factor that negatively impacts factors such as burnout (Arnold et al., 2015), turnover and retirement (Parasuraman and Alutto, 1984), and the wellbeing of home life, which is a private space. Given that employees’ creativity is a key factor in the innovation and competitiveness of an organization, the negative impact of stress on creative problem-solving performance indicates the urgency of the need for efforts to manage stress at an organizational level. Since it is known that acute stress increases risky decision-making by an increase of stress-induced dopamine in areas involved in reward processing and decision-making (Uy and Galván, 2017), organizational management efforts to identify and address individual-level stressors may reduce the intensity of stress that adversely affects employees’ physiological and cognitive functions. Recently, because ECG functions are added on wearable devices (e.g., Apple and Samsung Watch), allowing for the real-time measurement of HRV stress level and because low-cost, high-quality wearable brain wave measuring equipment (e.g., MUSE and Emotiv) is becoming rapidly generalized these days, this study may provide practical insights related to supporting employees under stress by leveraging the latest technologies at the organizational level.

### Limitations and Future Research Directions

The limitations of the present research, as well as possible future research directions, are as follows. First, this study considered stress to represent negative valence and positive arousal related to individual emotions (Giannakakis et al., 2019) to comprehensively reflect various job stressors (time pressure, role conflict, role ambiguity, etc.) experienced by individuals in organizational settings. In particular, the study used the money game as an attempt to induce stress among participants. However, because the induced stress herein may differ from the stress experienced in an actual management environment and because the degree and individual psychological evaluation of stress may vary depending on the type of stress, caution must be exercised when generalizing about work stress. Second, we carried out the experiment between 10:00 and 15:00 in the weekdays. However, the individual’s stress level can vary depending on the time of day. In general, the previous studies reveal that cortisol levels are highest in the early morning (Lindholm et al., 2012). Third, the participants comprised university students who were majoring in business management. This cannot exclude the possible effects of the particular characteristics of a limited group on the results herein. Fourth, owing to the constraints of the experimental environment, the problem-solving scenarios presented to the participants for the performance of the cognitive mapping test consisted of relatively simple content. Fifth, it is possible that the results of this research might be distorted because of the low sample size (i.e., recent recommendation of EEG experiments with between-subject need more than 25 samples for each condition) and non-direct stress-controlled creativity task. Lastly, the physiological data acquired after the stress-inducing game was compared between the groups in the study. Because the baseline before the game and the data after stress induction were not compared, it may not be clear whether the cause of the results of this study is stress. Therefore, future studies should utilize more realistic creativity tests with real business employees to identify the relationships between stress and problem-solving creativity with more samples and under the stress-controlled creativity task. In addition, it would be interesting to compare the variation in EEG and ECG signal between stress task and cognitive mapping task with in each group in the future research.

A detailed investigation of business problem-solving creativity is necessary wherein the job stressors that business workers experience is specified, and the differences in physiological responses by using more direct measurements such as cortisol measurements, GSR measurements, etc. In addition, it will be necessary to identify the activated brain regions related to creativity and stress during business problem-solving tasks by using neuroimaging methods such as fMRI and PET.

## Data Availability Statement

The original contributions presented in the study are included in the article/supplementary material, further inquiries can be directed to the corresponding author.

## Ethics Statement

This study was conducted with the approval of Sungkyunkwan University, in compliance with the guidelines and regulations of the university institutional review board (IRB no. 2017-12-011-022) for the method. The patients/participants provided their written informed consent to participate in this study.

## Author Contributions

JL designed the experiment, collected and analyzed the data, drafted, and revised the manuscript. CK assisted with the experiment, analyzed the data, drafted, and revised the manuscript. KL supervised the experimental design and the data collection and revised the manuscript. All authors contributed to the article and approved the submitted version.

## Funding

This work was supported by the National Research Foundation of Korea (NRF) grant funded by the Korea Government (MSIT; No. 2020R1F1A1074808).

## Conflict of Interest

The authors declare that the research was conducted in the absence of any commercial or financial relationships that could be construed as a potential conflict of interest.

## Publisher’s Note

All claims expressed in this article are solely those of the authors and do not necessarily represent those of their affiliated organizations, or those of the publisher, the editors and the reviewers. Any product that may be evaluated in this article, or claim that may be made by its manufacturer, is not guaranteed or endorsed by the publisher.
